# Mortality Prediction Model of Septic Shock Patients Based on Routinely Recorded Data

**DOI:** 10.1155/2015/761435

**Published:** 2015-10-18

**Authors:** Marta Carrara, Giuseppe Baselli, Manuela Ferrario

**Affiliations:** Department of Electronics, Information and Bioengineering, University Politecnico of Milan, Piazza Leonardo da Vinci 32, 20133 Milan, Italy

## Abstract

We studied the problem of mortality prediction in two datasets, the first composed of 23 septic shock patients and the second composed of 73 septic subjects selected from the public database MIMIC-II. For each patient we derived hemodynamic variables, laboratory results, and clinical information of the first 48 hours after shock onset and we performed univariate and multivariate analyses to predict mortality in the following 7 days. The results show interesting features that individually identify significant differences between survivors and nonsurvivors and features which gain importance only when considered together with the others in a multivariate regression model. This preliminary study on two small septic shock populations represents a novel contribution towards new personalized models for an integration of multiparameter patient information to improve critical care management of shock patients.

## 1. Introduction

The management of hemodynamic stability in shock patients is of paramount importance in critical care. Sepsis and septic shock are among the main reasons for intensive care unit (ICU) admission and they account for one of the highest mortality rate in noncoronary ICU (about 50%) [1]. Important medical societies, such as the European Society of Intensive Care Medicine and the Society of Critical Care Medicine, dedicated great attention to this topic by fostering debates and campaigns for developing new clinical guidelines (e.g., “Surviving Sepsis Campaign”).

An effective therapy is still lacking and there are no clear clinical signs either able to guide the right therapy or able to predict patient progress and outcome. The same definition of sepsis has been recently revised [2]. In 1991 sepsis was defined as the host's inflammatory response to infection, specifically by the presence of systemic inflammatory response syndrome (SIRS) criteria plus an infection. For simplicity, SIRS was defined by four variables: temperature, heart rate, respiratory rate, and white blood cell count. Only minor abnormalities in these variables are needed for a patient to meet these criteria [3]. Vincent et al. [2] highlight important weaknesses of this definition of sepsis. Indeed, the “softness” of SIRS criteria implies that up to 90% of the patients admitted to ICU meet these criteria even in cases when SIRS is caused by noninfectious clinical causes such as severe trauma, burns, pancreatitis, ischemic reperfusion events, or other forms of tissue injury that are accompanied by cell necrosis. From a molecular perspective, the initial host response to infection does not differ appreciably from the host response to sterile inflammation. Moreover, the host response to infections has beneficial aspects and a reduced or absent reaction of the subject is a symptom of other important diseases, such as immunodepression. For this reasons the authors proposed a new definition of sepsis as the host's deleterious, nonresolving inflammatory response to infection that leads to organ dysfunction [2].

Sepsis appears as a very complex and heterogeneous syndrome. The type of pathogen causing the infection, the pathogen burden, and the anatomic site together with the variety of responses of the host to the infection and the influence of existing comorbidities and age are all independent factors, which contribute to defining the heterogeneous nature of sepsis and septic shock. Early diagnosis, severity assessment, risk stratification, and mortality prediction of septic patients remain unsolved and the search for more effective therapies is still a major challenge for physicians.

In the very recent years, the enormous heterogeneity of sepsis syndrome has pushed researchers to adopt the concept of “personalized medicine” [4] which mainly refers to a biomarker-guided therapy. The large involvement of organs and cell systems in the inflammatory response has widened the number of possible candidates and many new biomarkers are being explored. Some of them are already in use in clinical settings: C-reactive protein (CRP) and procalcitonin (PCT), synthesized in the acute phase of sepsis, are routinely used as complementary tools in clinical decision-making. Beyond the acute phase proteins, a wide range of promising substances and nonlaboratory tools with potential diagnostic and prognostic value is under intensive investigation [5].

Multiple organ failure (MOF) is the fatal end of sepsis progression and it dramatically increases morbidity and mortality. Since the underlying mechanism, which leads to organs dysfunction, is not fully understood yet, researchers are exploring new biomarkers of endothelial integrity which is thought to play a fundamental role in the failure process. Moreover, nonlaboratory biomarkers, as the assessment of body temperature, heart rate variability, and cardiovascular parameters, can assist the clinicians in diagnosis, outcome monitoring, and prediction of septic patients [6–9].

The possibility to investigate the progression of shock with a larger integration of information at different scales and levels, such as at the molecular or cellular scale and at tissue or organ level by collecting hemodynamic signals and vital signs, would help in understanding the pathological mechanisms of the disease. Personalized models, based on this data integration, could be the basis for new more effective therapies and for preventing the development of shock in critical care patients.

In this work, we selected data from septic shock patients from the MIMIC-II database, which is an open access clinical database [10]. The objective was to develop a prediction model of 7-day mortality from vital signs and parameters routinely collected during the first 48 hours after shock onset.

## 2. Materials and Methods

### 2.1. Patients

MIMIC-II (version 2.6) includes data of more than 30,000 patients admitted at the ICUs of Boston's Beth Israel Deaconess Medical Center between 2001 and 2007.

Firstly, we selected 803 adult patients, that is, patients older than 18 years of age at time of admission, at their first hospital and ICU admission, who experienced a septic shock (i.e., their medical record clearly reports a shock event): they have ICD-9 code equal to 785.52 (dataset I).

Secondly, we took into consideration also the criteria suggested in the work of Angus et al. [11] as many patients should not be admitted with septic shock as primary cause of ICU admission, that is, ICD-9 code equal to 785.52. In this case we want to take into account patients with different progress of pathological state or different clinical history. Therefore we created another dataset (dataset II) by selecting the medical records with all the ICD-9 codes relating to both bacterial or fungal infections and a diagnosis of acute organ dysfunction. In this case we obtained a total of 3,585 adult patients at their first hospital and ICU admission. The following data, related to patient ICU staying, were extracted:

(i) Parameters derived from continuous hemodynamic signals: systolic arterial blood pressure (SBP, mmHg), diastolic arterial blood pressure (DBP, mmHg), mean arterial blood pressure (MAP, mmHg), heart rate (HR, bpm), respiratory rate (RR, breath per minute), central venous pressure (CVP, mmHg), and cardiac output (CO, L/min).
(ii) Clinical parameters and laboratory exams: temperature (*T*, °C), arterial pH (units), creatinine (mg/dL), blood glucose (mg/dL), lactate (mmol/L), hematocrit (%), white blood cell count (WBC, cells/cmm), and oxygen saturation SpO_2_ (%).
(iii) Amount of fluids administered: volume of fluids (mL) delivered. The total intake was calculated including all the intravenous infusions given to the patient.
(iv) Outcome: date of death.

We analyzed patients with at least 10 values of heart rate (HR), temperature (*T*), systolic blood pressure (SBP), respiratory rate (RR), and two values of white blood cells count (WBC). Moreover we excluded patients with ICU stay less than 48 hours. In dataset I, 713 patients out of 803 were excluded because they did not fulfill these inclusion criteria and 90 only were considered for further analyses. In dataset II only 545 out of 3,585 patients met these criteria.

### 2.2. Septic Shock Onset Detection

By considering the sequences of available data, we firstly identified time intervals which meet the four criteria for SIRS: (1) temperature > 38°C or < 36°C; (2) heart rate > 90 bpm; (3) respiratory rate > 20 breaths per minute; and (4) white blood cells count > 12,000 cells/cmm or < 4,000 cells/cmm. In particular, (i) the time intervals which meet the SIRS criteria must exceed 5 hours to be taken into consideration; (ii) if two or more intervals with abnormalities are less than 6 hours apart they are merged together to form a single episode; and (iii) the start of abnormality of the patient record corresponds to the time of the first measured parameter which met SIRS criteria. For example, if a patient has a temperature > 38°C from hour 1 to 23 and the first WBC was taken at hour 6 and it is abnormal, then abnormality interval starts at hour 1 and not at hour 6.

Successively, we identified septic shock onset according to the approach proposed in [1]; that is, a shock episode was defined as every time interval containing a SIRS episode where low SBP persists despite adequate fluid resuscitation. We identified all the intervals containing a prolonged hypotension, that is, with a SBP lower than 90 mmHg for at least 30 minutes. The total fluid intake was calculated starting one hour prior to the identified hypotension episode to halfway through the hypotension region. If total fluid intake was larger than 600 mL then that episode was classified as sepsis-induced hypotension. If more than one interval was classified as a sepsis-induced hypotension, the first one was labeled as shock onset. In case only one prolonged hypotensive episode was identified, we considered that record without septic shock episode as suggested in [1].

Only 23 out of 90 patients (~25%) and 73 out of 545 patients (~13%) showed a clear shock onset according to the criteria previously described and were used in final datasets I and II, respectively. Dataset II includes 21 patients from the 23 of dataset I.

### 2.3. Univariate and Multivariate Analysis

For each patient, we extracted hemodynamic, laboratory, and clinical data of the first 48 hours following the shock onset. For each of the data series previously described, we computed statistical indexes and we derived parameters relating to the series trend: mean, standard deviation, minimum and maximum values, median, kurtosis, skewness, regression slope of the series, and variation between the start and the end of the series (delta). Totally, we obtained 135 indexes.

The patients were subdivided into survivors (S) and nonsurvivors (NS) patients if they died within 7 days after the shock onset. The successive statistical analyses were performed on the two datasets separately.

S and NS groups were compared with the Wilcoxon Rank-Sum Test. The False Discovery Rate (FDR) was assessed as well due to the high number of comparisons. A *p* value < 0.05 was considered for the significance level.

We developed a prediction model of 7-day mortality from the 135 features by using dataset I and dataset II. We used a linear regression model with a new regularization and variable selection method named elastic net. This method was proposed by Zou and Hastie in 2005 [12] and it proved to outperform when the number of predictors *p* is much bigger than the number of observations* n*, in comparison with other regression models.

The best model was selected by using the 3-, 4-, and 5-fold cross-validation and by applying the one-standard error rule to the misclassification error. In order to avoid multicollinearity, the Variance Inflation Factor (VIF) was calculated and, iteratively, the features with VIF > 5 were excluded; VIF was then recalculated until all the values were under the threshold.

We evaluated the performance of the mortality model on dataset II only. We computed a linear regression model by using the features selected by the elastic net and a 5-fold cross-validation so as to compute the Area Under the Curve (AUC).

We adopted the mean imputation approach in order to deal with missing data, which were however a low percentage (8.1% for dataset I, 7.03% for dataset II).

Finally, we compared this prediction model with the traditional scores for mortality risk assessment in ICU; in this work we used the Sequential Organ Failure Assessment, SOFA score, and the Simplified Acute Physiology Score, SAPS I, the only available in the MIMIC-II database.

## 3. Results

**Figure 1:**
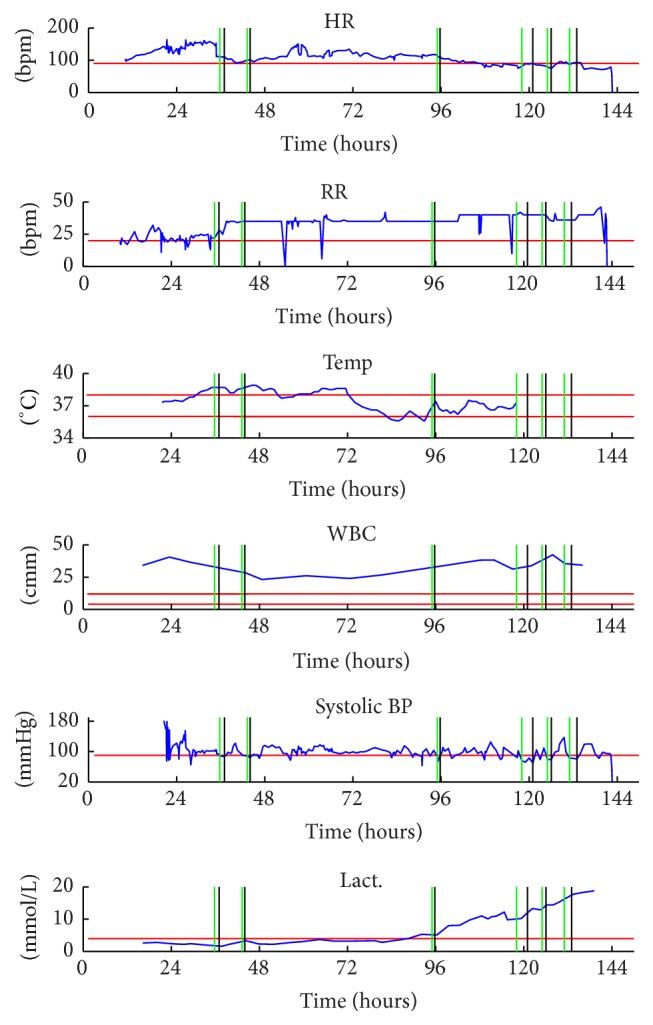
Example of parameters series from one patient (ID 6471). Green and black lines mark the start and the end of a shock episodes, respectively. The red lines indicate the threshold value for abnormality according to the SIRS criteria. After 6 days in ICU the patient dies. Notice that the values of lactate monotonically increase hinting organ dysfunction, hypoperfusion, and tissue injury. The values for WBC are maintained clearly over the threshold during the entire ICU staying, sign of a systemic inflammatory response. Observing the trend for the RR series, it is clearly visible that there is a sharp increase of the values till a plateau, synchronous with the shock onset. The patient receives about 1200 mL of fluids but he shows a persistent hypotension despite fluid resuscitation.

Figure 1 shows the time series of the vital signs in one patient and the shock onset is marked.

**Figure 2:**
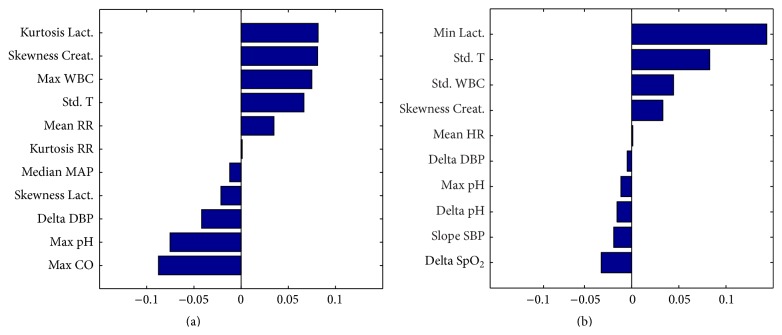
Barplot representation of the coefficients of the features selected from the elastic net regression model for dataset I and dataset II, in the upper and lower window, respectively.

Table 1 shows the features which are significantly different between survivors and nonsurvivors in dataset I.  Table 2 reports the results of univariate analysis on dataset II which are significant in addition to those already found in dataset I (see Table 1): standard deviation WBC, standard deviation *T*, mean SBP, median SBP, mean DBP, median DBP, mean MAP, median MAP, mean pH, maximum pH, median pH, mean SpO_2_, minimum SpO_2_, slope SBP, slope DBP, slope MAP, slope SpO_2_, delta SBP, delta DBP, delta MAP, delta pH, and delta SpO_2_. Tables 3 and 4 show the values of the coefficients of the features selected from the elastic net regression model for datasets I and II, respectively, and a graphical representation of them is also given in Figure 2. For each feature the corresponding VIF value is reported as well, and the mean square error of the models is annotated in the table headings.

AUC values obtained are the following: SOFA score: 0.74 ± 0.17, SAPS I: 0.95 ± 0.04, and proposed model: 0.97 ± 0.03.

## 4. Discussion

In this study we presented preliminary analyses on two small subsets of septic shock patients extracted from MIMIC-II database. Dataset I consists of only 23 septic shock subjects selected based on the presence of the specific ICD-9 code for septic shock. The second dataset includes 73 patients with both ICD-9 codes for fungal or bacterial infections and acute organ dysfunction, based on the criteria defined by Angus et al. [11].

The results from univariate and multivariate analysis identified features which play an important role in mortality prediction after shock onset. As expected, nonsurvivors have significant lower BP, decreased cardiac functionalities (described by low cardiac output (CO)), and reduced blood pH and oxygenation. The results obtained studying the trend of the physiological variables, that is, the slope and delta indices, could be interpreted as a lack of recovery by nonsurvivors. Blood pressure keeps on decreasing during the first 48 hours after the onset of a shock episode in NS patients, together with oxygen saturation (see Table 1), leading to organ dysfunction and death.

Some features that were not significantly different between S and NS in dataset I were, however, selected as important in univariate analysis on dataset II or, anyway, they gain more importance. It is the case, for example, of heart rate (HR), respiratory rate (RR), lactate and central venous pressure (CVP) (see Tables 1 and 2). On the other side, creatinine, whose distribution was found as significant in the first analysis, was not selected in the analyses in the larger dataset. These results could be explained by the fact that an increase of patients number permits more reliable estimates, but it rises the heterogeneity of the population, represented, for instance, by the different progress of patient condition or different pathological state at the ICU admission.

Some of the features that were found significant in the univariate analysis for discriminating S from NS are well known in literature as crucial to assess the patient's status. For example, hyperlactatemia is widely considered a symptom of poor outcome in ICU [13, 14] and creatinine is a well-established measure of renal activity and high levels in the blood are associated with severe renal dysfunction. An interesting consideration concerns the role of central venous pressure (CVP). The importance and the role of this physiological measure in critical care settings are still under debate. Guidelines recommend increased values of CVP as the end point of fluid resuscitation, based on the hypothesis that CVP reflects intravascular volume; that is, patients with low CVP are volume depleted whereas patients with high CVP are volume overloaded. However, recent studies demonstrated a poor relationship between CVP values and circulating blood volume and a reduced ability of CVP to predict fluid responsiveness, coming up with the idea that CVP should not be used anymore to guide fluid management strategies [15, 16]. Furthermore, a linear association between higher mean CVP in the first 24 hours from admission and increasing risk of new or persistent acute kidney injury (AKI) in septic patients was demonstrated by Legrand et al. [17], suggesting a role of venous congestion in the development of AKI. The authors suggested a revision of the affirmed clinical paradigm such that high target of CVP may reduce the occurrence of AKI, as part of the multiple organ failure syndrome. From our analyses (Table 1) we found out that the distribution of CVP and lactate and creatinine values are significantly different between the two populations of survivors and nonsurvivors.

We can guess that the time distributions and trends of the indexes, more than punctual values, may play a crucial role in the assessment of patient's status and the prediction of disease progress and outcome.

Not all the features selected in the multivariate analysis were also significant individually. We can notice that respiratory rate for dataset I and creatinine for dataset II were not significantly different between survivors and nonsurvivors, but, in the multiparameter model, they do have an impact on the model outcome.

Comparing Tables 3 and 4, relating to the multivariate model coefficients, lactate plays an important role in the model. For dataset I (Table 3), respiratory rate and cardiac output contribute to the final decision together with the other variables selected also in dataset II, while for dataset II (Table 4) heart rate and oxygen saturation play important role in the assessment of mortality risk.

These findings support a complex interdependence among different physiological systems in response to sepsis and septic shock. This reciprocal influence is at the basis of the big heterogeneity of the disease and a more detailed study of it could allow a risk stratification of the patients with effective incidence in early therapies. An example could be found in a recent work proposed by Knox et al. [18]. They present a clusterization of septic patients based on different combinations and burden or organ failure and they demonstrate a direct association of the clusters with 30-day risk of mortality. Using Self-Organizing Map (SOM) neural network technique they were able to identify four clusters of patients: cluster 1 that contains shock patients with elevated creatinine, cluster 3 that has shock patients with hypoxemia and altered mental status, while patients with severe sepsis were mostly in cluster 2 (minimal multiple organ dysfunction syndrome (MODS)), and cluster 4 (hepatic disease). Surprisingly, these results do not mirror the traditional classification of septic patients based on severity scores: elevated mortality was found in association with cluster 4 (severe sepsis with hepatic disease), whereas septic shock patients with elevated creatinine had lower mortality similar to patients with severe sepsis and minimal MODS.

Finally, AUC analyses support our study as the higher value is achieved by the proposed model (0.97 ± 0.03) followed by SAPS I (0.95 ± 0.04) and, last, SOFA score with a value of AUC under 0.8. Although some of the variables included in our model are the same as in the calculation of the scores (e.g., creatinine, blood pressure, heart rate, temperature, and WBC), the given model is proved to outperform traditional scores. Unlike SAPS and other ICU mortality scoring systems, SOFA was originally designed to focus more on organ dysfunction and morbidity, with less emphasis on mortality prediction, and this could be the reason why the performance of SOFA mortality prediction model was found to be lower. On the other side, SAPS reached a very good discrimination ability between S and NS, despite being overcome by the multivariate model. We could think that this gap is mainly due to a substantial difference in the approach: SAPS takes into account only the worst value of each variable over the past 24 hours for the computation, whereas our approach allows involving also trends and time distributions information of the values rather than single absolute measures.

## 5. Conclusion

The analyses show how the available information in a common ICU setting can be used to predict the progress of septic shock also in a very limited number of cases.

Vital signs available from measurements or their estimates are currently used for monitoring purposes in ICU. They convey system-wide, instantaneous information on the cardiovascular status of the patient, but they do not provide any insight into the fundamental mechanisms of disease. For this reason, in the recent years' clinical research is moving towards the study of new potential specific targets and biomarkers of sepsis and septic shock, trying to mark the root cause of the disease [19]. Discovering relationships and associations between bits of information at different physiological scales is thought to be the turning point in sepsis detection and early treatment. In this work we incorporated in the same model features derived from continuous hemodynamic monitoring, clinical parameters, and laboratory results and we demonstrated the validity of this approach in the mortality prediction problem. The huge quantity of multidimensional data collected by modern ICU is continuously increasing, calling for the need of new models for data integration.

## Figures and Tables

**Table 1:** Median (25°, 75°) values of the features significantly different between groups, dataset I.

Parameters	Survivors (15 pts.)	Nonsurvivors (8 pts.)	*p* value	FDR
Std. WBC (cells/cmm)	3.7 (2, 4.5)	6.2 (6, 7)	0.032	<0.01
Max WBC (cells/cmm)	18 (14.6, 29)	34.8 (31.6, 37.8)	0.035	<0.01
Std. *T* (°C)	0.4 (0.4, 0.6)	0.8 (0.5, 1)	0.026	<0.01
Min *T* (°C)	37 (35.8, 37.5)	35.5 (35.3, 36)	0.049	<0.01
Mean SBP (mmHg)	96 (94.7, 101.3)	91.2 (85.8, 97)	0.049	0.013
Median SBP (mmHg)	95 (94, 102)	88 (85, 94.3)	0.030	0.034
Mean DBP (mmHg)	59 (53.7, 66.2)	51.6 (49, 54.8)	0.015	<0.01
Median DBP (mmHg)	60 (54.5, 66.3)	52 (49, 54)	0.019	<0.01
Mean MAP (mmHg)	75 (69.4, 78.6)	64 (60.8, 67)	<0.01	<0.01
Median MAP (mmHg)	75 (67, 77.5)	64 (60.8, 66.5)	<0.01	<0.01
Kurtosis lactate	2.2 (1.7, 2.5)	3 (2.6, 3.2)	0.039	<0.01
Std. CO (L/min)	1.2 (0.8, 1.6)	0.7 (0.4, 0.8)	0.049	<0.01
Max CO (L/min)	7.9 (7.3, 11.3)	5.8 (4.4, 6.8)	<0.01	<0.01
Skewness CVP	0.5 (0.1, 1.3)	0 (−0.5, 0.2)	0.024	<0.01
Mean pH (units)	7.3 (7.3, 7.4)	7.2 (7.2, 7.3)	<0.01	<0.01
Max pH (units)	7.4 (7.4, 7.5)	7.3 (7.3, 7.3)	<0.01	<0.01
Median pH (units)	7.3 (7.3, 7.4)	7.2 (7.2, 7.3)	0.016	<0.01
Mean SpO_2_ (%)	97.3 (96.6, 97.7)	94.4 (90.5, 96)	<0.01	<0.01
Min SpO_2_ (%)	89 (75.5, 92.8)	62.5 (15, 88)	0.024	0.099
Skewness Creatinine	−0.2 (−0.6, 0.2)	0.5 (0.2, 0.6)	0.032	<0.01
Slope SBP (mmHg/h)	0.33 (0.28, 0.37)	−0.04 (−0.22, 0.12)	0.018	<0.01
Slope DBP (mmHg/h)	0.14 (−0.05, 0.26)	−0.13 (−0.55, 0.04)	0.015	<0.01
Slope MAP (mmHg/h)	0.23 (0.03, 0.33)	−0.15 (−0.78, 0.06)	<0.01	<0.01
Slope SpO_2_ (%/h)	0 (−0.07, 0.03)	−0.19 (−0.67, −0.07)	<0.01	<0.01
Delta SBP (mmHg)	24 (11, 34.7)	−2 (−36, 11.5)	<0.01	<0.01
Delta DBP (mmHg)	10 (5, 19)	−6.5 (−21.5, 3.5)	<0.01	<0.01
Delta MAP (mmHg)	15 (8, 20.7)	−4 (−19.5, 4.5)	<0.01	<0.01
Delta pH (units)	0.09 (0, 0.17)	−0.05 (−0.11, 0.03)	0.016	<0.01
Delta SpO_2_ (%)	0 (−1, 3)	−2 (−20, −0.5)	0.028	<0.01

WBC = white blood cell count. *T* = temperature. SBP = systolic blood pressure. DBP = diastolic blood pressure. MAP = mean arterial pressure. CO = cardiac output. CVP = central venous pressure. Std. = standard deviation.

**Table 2:** Median (25°, 75°) values of the features significantly different between groups, dataset II (only the features which are not already shown in Table 1).

Parameters	Survivors (53 pts.)	Nonsurvivors (20 pts.)	*p* value	FDR
Mean HR (bpm)	103.1 (93.6, 110.4)	115 (104, 124)	<0.01	<0.01
Std. HR (bpm)	11.8 (9.2, 16.8)	15.7 (10, 24)	0.046	0.058
Median HR (bpm)	102 (90.1, 111)	112.2 (102.5, 124.5)	<0.01	0.01
Mean RR (breaths/min)	22.8 (19, 25.6)	24.9 (23.3, 27.2)	0.025	0.036
Median RR (breaths/min)	24 (19, 26)	25.5 (24, 30)	0.014	0.025
Max *T* (°C)	38.3 (37.6, 38.8)	38.9 (37.9, 39.5)	0.049	0.062
Kurtosis *T*	2.5 (1.9, 3.2)	2.1 (1.6, 2.2)	0.035	0.047
Max SBP (mmHg)	144 (128, 157)	129.5 (116, 150.5)	0.046	0.06
Min MAP (mmHg)	51 (44.2, 56.2)	39 (17, 52.5)	0.018	0.027
Mean lactate (mmol/L)	3.2 (1.7, 5.1)	6.7 (3.8, 12.7)	<0.01	<0.01
Min lactate (mmol/L)	1.8 (1.4, 2.5)	5.15 (2.5, 7.8)	<0.01	<0.01
Max lactate (mmol/L)	4.8 (2.45, 7.37)	8 (5.3, 16.9)	0.014	0.024
Median lactate (mmol/L)	3 (1.7, 5)	6.8 (3.8, 11.9)	<0.01	<0.01
Mean CVP (mmHg)	14.3 (12.1, 18.5)	17.3 (16.3, 19.9)	0.012	0.023
Min CVP (mmHg)	8 (4, 11)	11 (9, 15)	<0.01	0.011
Median CVP (mmHg)	15 (11.7, 17)	17 (16, 19)	<0.01	0.016
Min pH (units)	7.25 (7.17, 7.35)	7.15 (7.08, 7.21)	<0.01	<0.01
Skewness pH	−0.01 (−0.5, 0.4)	−0.61 (−0.91, −0.12)	0.028	0.039
Slope pH (units/h)	0 (−0.001, 0.004)	−0.001 (−0.004, 0)	<0.01	0.014
Std. SpO_2_ (%)	2.7 (1.7, 4.4)	5.5 (2.1, 11)	0.028	0.039
Median SpO_2_ (%)	97 (96, 99)	95.75 (94.5, 97.5)	0.017	0.026

HR = heart rate. RR = respiratory rate. *T* = temperature. SBP = systolic blood pressure. MAP = mean arterial pressure. CVP = central venous pressure. Std. = standard deviation.

**Table 3:** Coefficients of the elastic net regression, dataset I (MSE is 0.0302).

Parameters	Coefficients	VIF
Maximum CO (L/min)^*∗*^	−0.0875	2.09
Maximum pH (units)^*∗*^	−0.0753	2.5
Delta DBP (mmHg)^*∗*^	−0.0418	1.78
Skewness lactate	−0.0214	2.39
Median MAP (mmHg)^*∗*^	−0.0121	2.14
Kurtosis RR	0.0012	2.12
Mean RR (breath/min)	0.0348	2.38
Std. *T* (°C)^*∗*^	0.0666	3.09
Maximum WBC (cells/cmm)^*∗*^	0.0749	1.63
Skewness creatinine^*∗*^	0.0811	2.39
Kurtosis lactate^*∗*^	0.0815	2.63

^*∗*^Significant features also in the univariate analysis.

**Table 4:** Coefficients of the elastic net regression, dataset II (MSE is 0.1095).

Parameters	Coefficients	VIF
Delta SpO_2_ (%)^*∗*^	−0.0323	1.6
Slope SBP (mmHg/h)^*∗*^	−0.0191	1.52
Delta pH (units)^*∗*^	−0.0154	1.63
Max pH (units)^*∗*^	−0.0114	1.48
Delta DBP (mmHg/h)^*∗*^	−0.0047	1.91
Mean HR (bmp)^*∗*^	0.0012	1.32
Skewness Creatinine	0.033	1.13
Std. WBC (cells/cmm)^*∗*^	0.0443	1.38
Std. *T* (°C)^*∗*^	0.0827	2.03
Min lactate (mmol/L)^*∗*^	0.1431	1.93

^*∗*^Significant features also in the univariate analysis.
